# Evidence that Ergosterol Biosynthesis Modulates Activity of the Pdr1 Transcription Factor in Candida glabrata

**DOI:** 10.1128/mBio.00934-19

**Published:** 2019-06-11

**Authors:** Bao Gia Vu, Grace Heredge Thomas, W. Scott Moye-Rowley

**Affiliations:** a Department of Molecular Physiology and Biophysics, Carver College of Medicine, University of Iowa, Iowa City, Iowa, USA; Universidade de Sao Paulo

**Keywords:** *Candida glabrata*, Pdr1, Upc2A, azole resistance, ergosterol, gene regulation, transcription factors

## Abstract

A crucial limitation in antifungal chemotherapy is the limited number of antifungal drugs currently available. Azole drugs represent the most commonly used chemotherapeutic, and loss of efficacy of these drugs is a major risk factor in successful treatment of a variety of fungal diseases. Candida glabrata is a pathogenic yeast that is increasingly found associated with bloodstream infections, a finding likely contributed to by its proclivity to develop azole drug resistance. C. glabrata often acquires azole resistance via gain-of-function (GOF) mutations in the transcription factor Pdr1. These GOF forms of Pdr1 drive elevated expression of target genes, including the ATP-binding cassette transporter-encoding *CDR1* locus. GOF alleles of *PDR1* have been extensively studied, but little is known of how Pdr1 is normally regulated. Here we test the idea that reduction of ergosterol biosynthesis (as occurs in the presence of azole drugs) might trigger activation of Pdr1 function. Using two different means of genetically inhibiting ergosterol biosynthesis, we demonstrated that Pdr1 activity and target gene expression are elevated in the absence of azole drug. Blocks at different points in the ergosterol pathway lead to Pdr1 activation as well as to induction of other genes in this pathway. Delivery of the signal from the ergosterol pathway to Pdr1 involves the transcription factor Upc2A, an *ERG* gene regulator. We show that Upc2A binds directly to the *PDR1* and *CDR1* promoters. Our studies argue for a physiological link between ergosterol biosynthesis and Pdr1-dependent gene regulation that is not restricted to efflux of azole drugs.

## Abstract (precis)

A likely contributor to the increased incidence of non-*albicans* candidemias involving Candida glabrata is the ease with which this yeast acquires azole resistance, in large part due to induction of the ATP-binding cassette transporter-encoding gene *CDR1*. Azole drugs lead to induction of Pdr1 transactivation, with a central model being that this factor binds these drugs directly. Here we provide evidence that Pdr1 is activated without azole drugs by the use of genetic means to inhibit expression of azole drug target-encoding gene *ERG11*. These acute reductions in Erg11 levels lead to elevated Pdr1 activity even though no drug is present. A key transcriptional regulator of the *ERG* pathway, Upc2A, is shown to directly bind to the *PDR1* and *CDR1* promoters. We interpret these data as support for the view that Pdr1 function is responsive to ergosterol biosynthesis and suggest that this connection reveals the normal physiological circuitry in which Pdr1 participates.

## INTRODUCTION

Invasive candidiasis is caused primarily by Candida albicans, but a recent trend is the disturbing increase in infections caused by non-*albicans* species (1–3). Candida glabrata is the second most common species associated with candidiasis, and infections by these species are associated with increasingly common reduced antifungal susceptibility. The limited number of distinct antifungal drug classes makes resistance a serious threat to continued effective chemotherapy (reviewed in reference 4).

The most commonly used antifungal drug class is represented by azole compounds. Anti-*Candida* chemotherapy routinely utilizes fluconazole, a drug that can be administered orally and that has good selectivity for the target enzyme of the pathogen, lanosterol α-14 demethylase (recently discussed in reference 5). This enzyme is encoded by the *ERG11* gene in the *Candida* genera. The *ERG11* gene is essential for production of the fungal sterol ergosterol, a critical component of the fungal plasma membrane. Loss of *ERG11* is a lethal event or causes a profound growth defect in most *Candida* species (6–8).

Resistance to fluconazole is most commonly associated with single amino acid substitution mutations in the gene encoding a Zn_2_Cys_6_ zinc cluster-containing transcription factor called Pdr1 (recently reviewed in reference 9). These mutations yield a gain-of-function (GOF) phenotype and lead to the elevated transcription of downstream target genes. The ATP-binding cassette (ABC) transporter-encoding *CDR1* gene is one of the principal targets of Pdr1 and is required for the elevated fluconazole resistance seen in such *PDR1* GOF mutant strains (10, 11). The GOF alleles of *PDR1* cause chronically increased transcription of downstream target genes through the enhanced ability to activate gene expression (12).

Experiments reported from several groups demonstrated that wild-type Pdr1 activity is responsive to challenge with fluconazole, leading to strong autoregulatory induction of *PDR1* itself as well as to activation of *CDR1* gene transcription (10–12). Both biochemical and genetic approaches were used to argue that azole drugs bind directly to Pdr1 and that this binding leads to activation of Pdr1 function (13).

An intrinsic complication of the use of fluconazole to induce Pdr1 function is its concomitant inhibition of ergosterol biosynthesis. We wanted to test if it were possible to separate the presence of fluconazole from a block in ergosterol production at the level of *ERG11*. To do this, we utilized two different repressible promoter systems that could be transcriptionally repressed via completely different means. Irrespective of how *ERG11* transcription was halted, this was followed by activation of Pdr1 and increased transcription of its target genes. We found that induction of Pdr1 target genes also required the ergosterol-regulated Upc2A transcription. Upc2A is required for normal expression of ergosterol biosynthetic genes. Strikingly, chromatin immunoprecipitation (ChIP) indicated that Upc2A was able to bind to a site in the *PDR1* and *CDR1* promoters, providing a direct link between ergosterol biosynthesis and a key determinant of azole resistance. Our data provide the first evidence tying control of Pdr1-dependent gene expression that impacts azole resistance to the activity of the biosynthetic pathway inhibited by azole drugs. We propose that coordinated control of ergosterol biosynthesis and of integral membrane proteins such as Cdr1 is required for regulation of permeability through the plasma membrane.

## RESULTS

### Fluconazole activates expression of Erg11, Cdr1, and Pdr1 at both the protein and mRNA levels.

Previous work has established that fluconazole challenge leads to a rapid and robust increase in transcription of a range of different genes involved in ergosterol biosynthesis, including *ERG11* genes as well as *PDR1* and its target genes such as *CDR1* (14, 15). To directly examine the link between mRNA levels and steady-state protein levels, we generated immunological probes against Cdr1 and Erg11 by raising a polyclonal antiserum or attaching a 3× hemagglutinin (HA) epitope, respectively, to these proteins. We have already described a rabbit antiserum that can detect Pdr1 (16). We inserted a 3× HA tag at the C-terminus of the *ERG11* gene and ensured that this epitope-tagged allele supported normal growth and azole susceptibility (data not shown). This strain was designated BVGC3. We grew BVGC3 to mid-log phase and then challenged this strain with fluconazole for various times. Whole-cell protein extracts were prepared and analyzed by Western blotting with the indicated antibodies.

**FIG 1:**
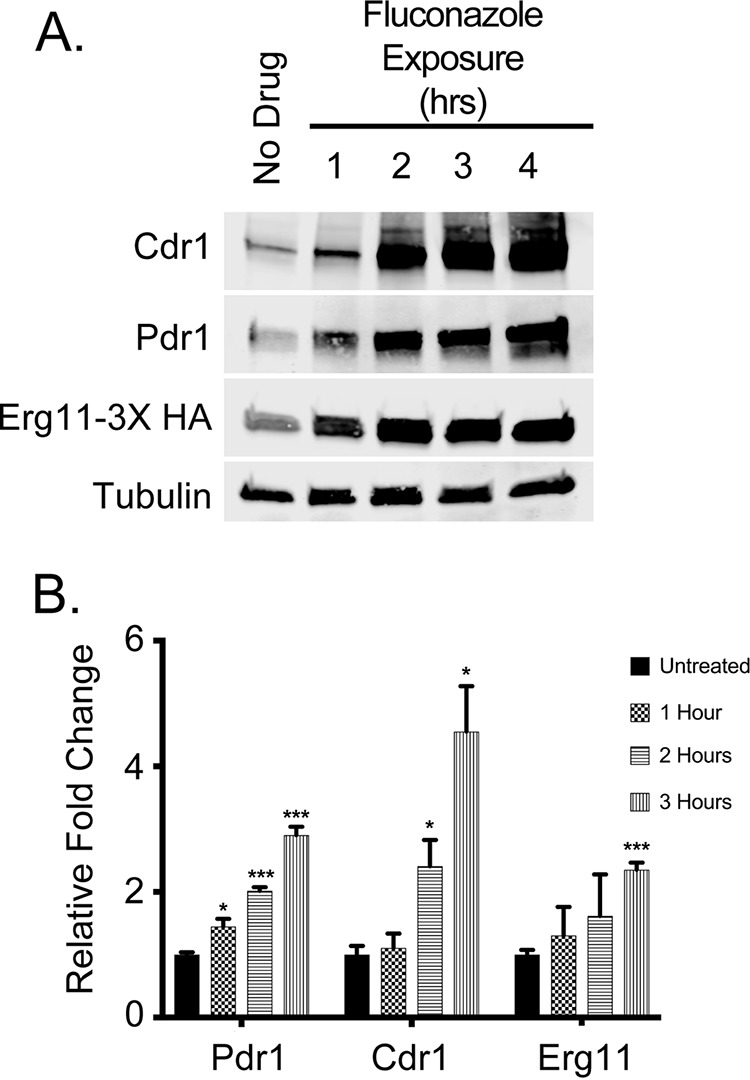
Western blot analysis of fluconazole induction of Cdr1, Pdr1, and Erg11. A C. glabrata strain containing an epitope-tagged version of *ERG11* (*ERG11*-3× HA) was grown to early log phase and then treated with 20 μg/ml fluconazole for the times indicated. An aliquot was withdrawn prior to drug challenge (No Drug) to provide a control for non-drug-stimulated levels. Whole-cell protein extracts were prepared and analyzed by Western blotting using rabbit antibodies against Cdr1 or Pdr1 and mouse monoclonal antibodies against the HA epitope (Erg11-3× HA) or β-tubulin. (B) The fold changes of Cdr1, Pdr1, and Erg11 levels were calculated by normalizing later time points to the initial protein level determined as described for panel A.

Cdr1, Pdr1, and Erg11 were all induced by fluconazole at the level of each protein, with induction easily seen at 1 h after drug exposure (Fig. 1A). Quantitation of these blots demonstrated that the fold induction level was somewhat greater for Cdr1 and Pdr1 but was more than 2-fold above the control level for Erg11 (Fig. 1B). Blotting for tubulin confirmed that the levels of loading were equal for these samples. These data confirm that fluconazole treatment induces these genes at both the transcriptional and translational levels.

### Development of an acutely repressible form of *ERG11*.

In order to quickly block *ERG11* expression, we adapted a camphor-regulated expression system developed in Saccharomyces cerevisiae for use in C. glabrata. This system is based on a fusion protein between the bacterial camphor repressor protein (CamR) and the strong viral transactivator protein VP16. CamR binds strongly to DNA until exposed to its substrate, camphor, a natural product (17). Thus, the CamR-VP16 activates expression of genes with the CamR binding site (camO) placed as an upstream activation sequence. Our goal was to develop a genetic means of inhibiting ergosterol biosynthesis that would allow us to evaluate if direct binding of fluconazole to Pdr1 was required for induction or if a reduction in Erg11 activity was sufficient.

**FIG 2:**
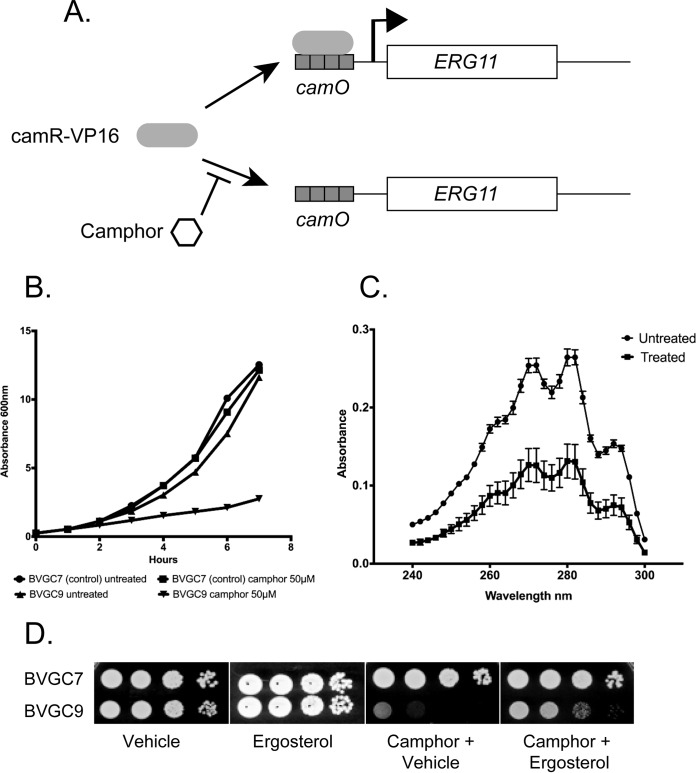
Characterization of camphor-regulated *ERG11* allele. (A) A diagram showing the control of the camR-regulated *camPr-ERG11* fusion gene. In the absence of camphor, camR-VP16 can bind to and induce *camPr-ERG11* expression. The presence of camphor causes camR-VP16 to be released from this promoter, and *ERG11* expression is strongly inhibited. (B) The presence of the *camPr-ERG11* allele confers a camphor-sensitive growth phenotype. Strains containing both Sc*TDH1*-driven camR-VP16 and *camPr-ERG11* (BVGC9) or only Sc*TDH1*-driven camR-VP16 (BVGC7) were grown overnight and then diluted into media containing or lacking camphor as indicated. Cultures were allowed to grow at 30μC, and optical density was measured using a spectrophotometer. (C) Mid-log phase cultures of BVGC9 grown in the absence (Untreated) or the presence (Treated) of 50 μM camphor for 2 h were harvested and ergosterol levels measured as described previously (41). (D) Cultures of the indicated strains were grown to the mid-log phase and then diluted for plating on rich media containing the listed additions. “Vehicle” represents the solution used to solubilize ergosterol.

We generated a C. glabrata strain that produced CamR-VP16 from the S. cerevisiae *TDH1* (Sc*TDH1*) promoter. This strain was designated BVGC7. To produce the camphor-repressible allele of *ERG11*, we replaced the *ERG11* promoter in BVGC7 with a multimerized camO element upstream of the Sc*CYC1* TATA region (camPr) to obtain BVGC9. This strain produced Erg11 in a camphor-sensitive manner (Fig. 2A).

We compared the levels of sensitivity of the isogenic BVGC7 and BVGC9 strains with respect to growth in the presence of camphor. Since *ERG11* is essential for normal growth in C. glabrata, we anticipated that inappropriate transcriptional repression would cause a growth defect when the only source of *ERG11* was the camphor-repressible promoter. This was found to be the case, as BVGC9 growth was quite sensitive to the presence of camphor (Fig. 2B). We also assayed ergosterol levels in BVGC9 cells grown for 2 h in the presence or absence of camphor. This assay showed that ergosterol levels were strongly reduced when cells were grown under camphor-repressing conditions (Fig. 2C).

Finally, we tested the camphor sensitivity of BVGC9 growth on solid yeast extract-peptone-dextrose (YPD) media. Serial dilutions of BVGC7 (transactivator only) and BVGC9 (both transactivator-driven and camO-driven *ERG11*) were plated on media with the vehicle used to solubilize ergosterol, with ergosterol, with camphor plus vehicle, or with camphor plus ergosterol (Fig. 2D). Growth of BVGC9 was sensitive to camphor, while BVGC7 grew normally. Addition of ergosterol along with camphor rescued growth of BVGC9 in the presence of camphor. These data are consistent with the view that the camO-Sc*CYC1*-*ERG11* fusion gene makes sufficient Erg11 enzyme to support growth of cells but does so only in the absence of camphor. To determine if expression changes of *PDR1* and *CDR1* would respond to diminished levels of Erg11 and to confirm that regulation of *ERG11* was consistent with the growth data, we measured both the mRNA and protein levels of these loci.

**FIG 3:**
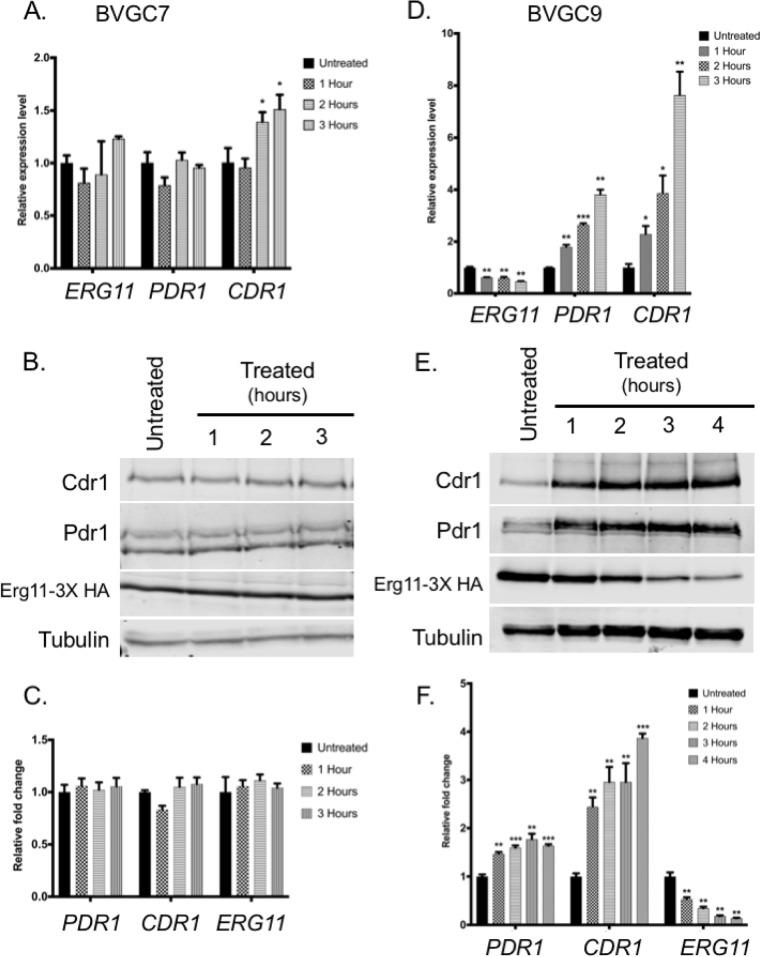
Both *PDR1* and *CDR1* are induced upon transcriptional inactivation of the *camPr-ERG11* fusion. (A) The strain containing only the camR-VP16 transactivator (BVGC7) was grown to the mid-log phase and then treated with 50 μM camphor for the indicated lengths of time. Total RNA was prepared from each culture at each time point. Levels of the indicated mRNAs were determined by qRT-PCR. (B) Whole-cell protein extracts were prepared from the indicated BVGC7 cultures, and then the levels of Pdr1, Cdr1, Erg11-3× HA, and tubulin were determined by Western blotting with appropriate antisera. (C) Quantitation of Western blots prepared as described in the panel B legend. (D) BVGC9 (which contains both transactivator-responsive and camphor-responsive *ERG11* alleles) was grown to the mid-log phase and treated with camphor to inactivate *ERG11* expression for various times. RNA levels of *ERG11*, *PDR1,* and *CDR1* were measured using qRT-PCR. (E) Whole-cell protein extracts were prepared from BVGC9 cultures treated with camphor for various times or left untreated. Western blot analyses were used to measure expression of the indicated proteins. (F) Quantitation of Western blots prepared as described for panel E.

BVGC9 was grown to the mid-log phase and then treated with 50 μM camphor for 1 to 4 h. Total RNA and whole-cell protein extracts were prepared at each time point for analysis by reverse transcription-quantitative PCR (qRT-PCR) and Western blotting, respectively. The presence of camphor led to a prompt and sustained suppression of *ERG11* mRNA in BVGC9 (Fig. 3D) but not in the strain containing only the camphor-dependent transactivator (BVGC7) (Fig. 3A). Transcription of both *PDR1* and *CDR1* was elevated similar time courses during camphor-mediated *ERG11* repression in BVGC9. These changes in mRNA levels were reflected in protein accumulation (Fig. 3E), as anticipated. Importantly, Erg11-3× HA levels were reduced by approximately 50% after the addition of camphor and continued to decline over the course of this treatment. Abundances of both Pdr1 and Cdr1 were elevated within an hour and remained at that level during the camphor treatment. Control experiments (Fig. 3A to C) performed using the transactivator-only strain (BVGC7) showed no significant changes in expression of any of these genes or their corresponding proteins in response to camphor. Minor changes in *CDR1* transcription were seen at later time points with camphor treatment but were not seen via Western blotting.

### Response of the *ERG* pathway to defects in *ERG11* transcription.

**FIG 4:**
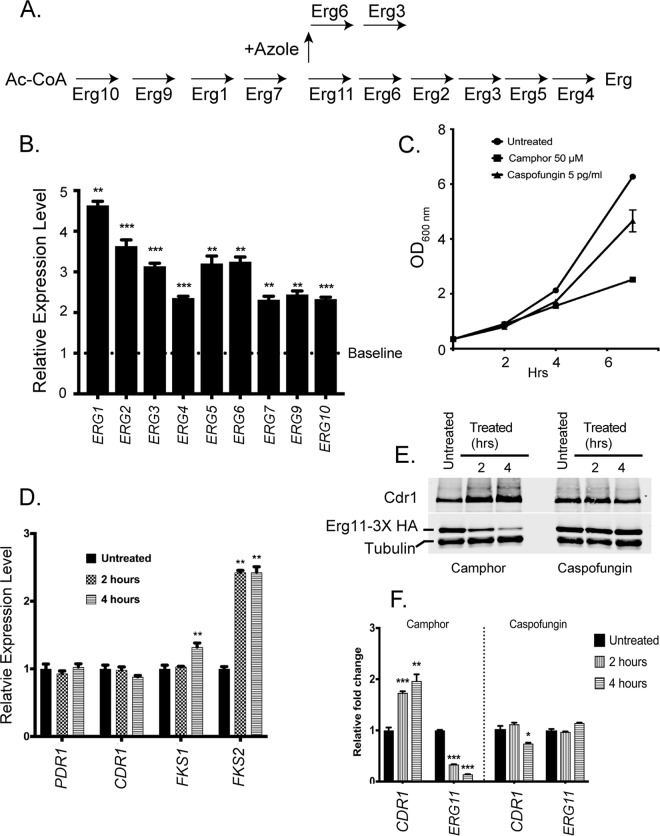
Effects of camphor-mediated transcriptional repression on the ergosterol pathway. (A) Relevant steps in the ergosterol biosynthetic pathway are indicated. The addition of azole drugs blocks Erg11 function and causes production of a toxic sterol. Ac-CoA, acetate coenzyme A. (B) BVGC9 cells were left untreated or grown in the presence of camphor for 3 h. Total RNA was prepared and levels of the indicated ERG pathway genes determined by qRT-PCR. Relative expression values were determined by normalizing the expression levels seen after camphor treatment to the level of each transcript produced in untreated cells (Baseline). (C) Caspofungin treatment at a subinhibitory level caused a growth defect similar to that seen after addition of camphor to BVGC9. (D) Caspofungin challenge induces *FKS2* transcription but not *PDR1* or *CDR1* transcription. Cells were grown with the echinocandin drug or left untreated for 2 or 4 h. Total RNA was prepared and levels of transcripts of interest assessed by qRT-PCR. (E) BVGC9 cells were grown as described for panel C and whole-cell protein extracts prepared at the indicated time points. Cdr1, Erg11-3× HA, and tubulin levels were determined by Western blot analysis. (F) Quantitation of the Western blot data in panel E.

While the data presented above demonstrate that loss of normal *ERG11* mRNA levels triggered *PDR1* and *CDR1* transcriptional increases, we wanted to determine if other genes in the *ERG* pathway were sensitive to *ERG11* mRNA levels. We selected 9 *ERG* genes (*ERG1*, *ERG2*, *ERG3*, *ERG4*, *ERG5*, *ERG6*, *ERG7*, *ERG9*, and *ERG10*) to represent the behavior of the rest of the pathway (see Fig. 4A). BVGC9 was grown to the mid-log phase and then treated with camphor for 2 h or left untreated. We isolated mRNA from these cultures and analyzed levels of the pathway genes by qRT-PCR. The differences in the gene expression levels seen under camphor-treated versus untreated conditions are presented as ratios (Fig. 4B).

Camphor inhibition of *ERG11* transcription led to at least 2-fold induction of all tested genes, with *ERG1* showing the greatest increase (up to 4-fold). The coregulation of these pathway genes would be expected from their necessary participation in the multistep biosynthesis of ergosterol, an essential metabolite for the cell.

Blocking ergosterol biosynthesis caused a clear growth inhibition such as has been demonstrated using a doxycycline-repressible allele of *ERG11* (7). To ensure that the transcriptional induction of *PDR1* and *CDR1* represents a response to loss of ergosterol biosynthesis and not to this particular form of growth inhibition, we used the β-glucan synthase inhibitor caspofungin as a different means of preventing normal growth. A sublethal dose (5 pg/ml) of caspofungin was used that led to a level of acute inhibition of BVGC9 similar to that elicited by camphor (Fig. 4C). To confirm that caspofungin caused the expected stress in β-glucan synthesis, mRNA levels for *FKS1* and *FKS2* were assayed. These genes encode the β-glucan synthase enzyme and represent the direct targets of caspofungin. *PDR1* and *CDR1* mRNA levels were also measured to compare their responses to this echinocandin. Total RNA was prepared from caspofungin-treated (2 and 4 h) cells or from untreated cells, and mRNAs of interest were quantitated by qRT-PCR (Fig. 4D).

Among these 4 genes, only *FKS2* was induced by treatment with caspofungin, as others have documented (18). This gene encodes the echinocandin-inducible form of β-glucan synthase. Notably, neither *PDR1* transcription nor *CDR1* transcription responded to this block in growth.

We also compared the response of Cdr1 and Erg11-3× HA to caspofungin-induced growth inhibition relative to that caused by camphor addition in BVGC9 (Fig. 4E and F). Caspofungin challenge that caused the observed induction of *FKS2* mRNA had no significant effect on the steady-state expression levels of either Cdr1 or Erg11-3× HA. Camphor treatment had the expected effect, leading to induction of Cdr1 and repression of Erg11-3× HA. These data support the interpretation that lowered levels of *ERG11* generate a signal leading to an induction of *PDR1* and *CDR1* transcription that extends beyond the inhibition of growth.

### Multiple blocks in the *ERG* pathway induce *PDR1* and *CDR1*.

**FIG 5:**
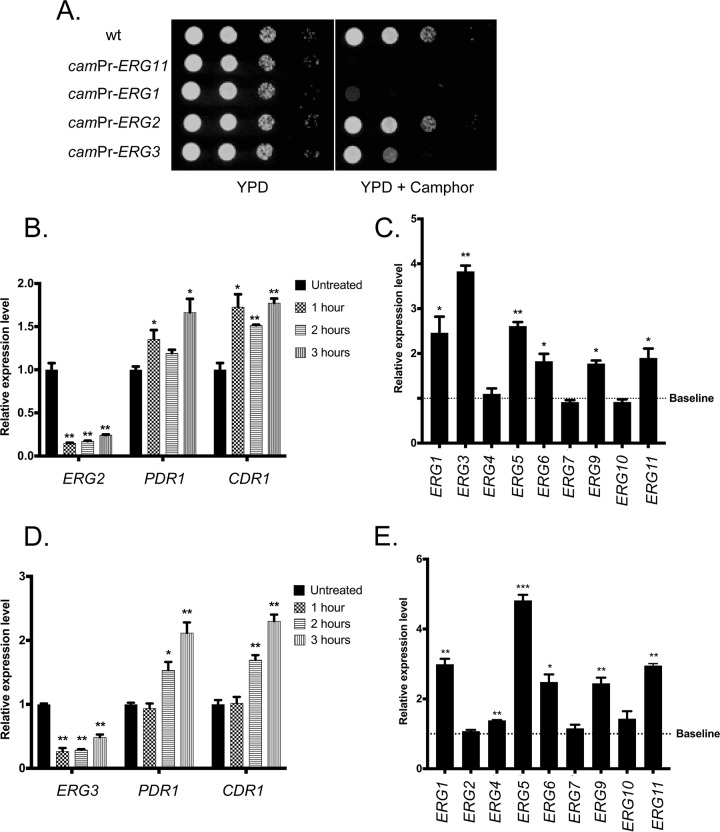
Camphor-off alleles of other *ERG* genes also lead to induction of *PDR1* and *CDR1*. (A) Three additional strains were produced that contained both the camR-VP16 transactivator and the camphor-off alleles of *ERG1*, *ERG2*, or *ERG3*. These strains, along with a wild-type control, BVGC9 (camR-VP16, camPr-*ERG11*) were grown to the mid-log phase and serial dilutions plated on rich medium (YPD) or YPD containing 50 μM camphor. Plates were then incubated at 30μC and photographed. (B) The camphor-repressible *ERG2* fusion gene-containing strain was grown and left untreated or challenged with camphor for the indicated times. Total RNA was prepared from each sample and analyzed for expression of *ERG2*, *PDR1*, and *CDR1* mRNA by qRT-PCR. (C) The transcriptional response of the *ERG* pathway genes was determined by qRT-PCR from cells grown as described for panel B. (D) The strain with the camphor-repressible *ERG3* gene was grown in the absence or presence of camphor as described above followed by the indicated qRT-PCR analyses. (E) The transcriptional responses of the *ERG* pathway to camphor-mediated *ERG3* repression determined across the indicated *ERG* pathway genes.

Having provided the evidence detailed above showing that camphor-mediated repression of *ERG11* transcription led to activation of *PDR1* and *CDR1* transcription, we constructed three other *ERG* gene promoter replacements. We selected *ERG1*, *ERG2*, and *ERG3* for this analysis. In each case, the wild-type promoter was replaced with the camphor-regulated form discussed above in a manner analogous to that used with *ERG11*. These three new strains were grown to the mid-log phase and then placed on solid rich medium in the presence or absence of camphor (Fig. 5A).

The *camPr-ERG1* gene was associated with strongly camphor-sensitive growth that was only slightly better than that seen with the BVGC9 strain. The *camPr-ERG3* strain was found to grow less well than the *camPr-ERG2* strain, which showed no growth reduction in the presence of camphor. Along with the growth phenotypes caused by camphor addition, we also compared the levels of expression of both the *PDR1*/*CDR1* system and the *ERG* pathway by qRT-PCR in all these strains.

Replacement of either *ERG2* (Fig. 5B) or *ERG3* (Fig. 5D) with camPr caused expression of the resulting fusion genes to be strongly repressed upon the addition of camphor, as expected. The modest reduction in growth of the camphor-repressed *camPr-ERG3* strain compared to the *camPr-ERG2* strain was likely due to accumulation of different ergosterol precursors in these two strains. Similarly, the levels of expression of both *PDR1* and *CDR1* were elevated upon camphor treatment in both strains. The levels of *ERG* pathway induction seen in these two strains were also generally similar (compare Fig. 5C and E), although camphor repression of the *camPr-ERG2* fusion strain triggered the largest induction of *ERG3* mRNA whereas similar treatment of the *camPr-ERG3* strain showed no effect on *ERG2* mRNA. The levels of expression of the *ERG4*, *ERG7*, and *ERG10* genes were not altered by repression of either *ERG2* or *ERG3*, while the *ERG1*, *ERG5*, *ERG6*, *ERG9*, and *ERG11* genes were all found to be induced. This suggests the presence of selective regulatory circuitry modulating the different *ERG* genes rather than a single common response.

**FIG 6:**
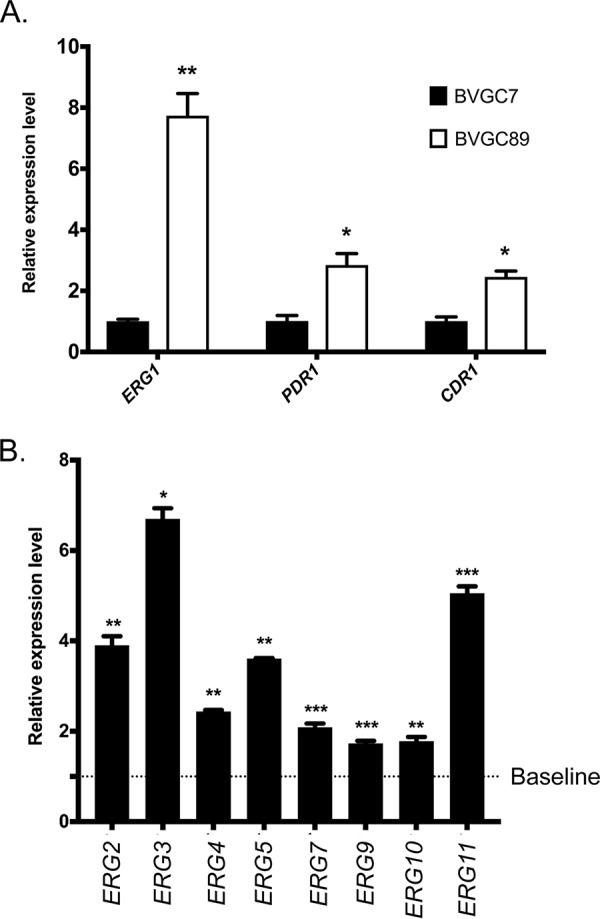
Elevated *ERG1* mRNA levels trigger induction of *PDR1*, *CDR1*, and *ERG* pathway genes. Strains containing a camphor-off allele of *ERG1* (BVGC89) or the camphor transactivator only (BVGC7) were grown to the mid-log phase, and cells were harvested and used to prepare total RNA. (A) Expression of *ERG1*, *PDR1*, and *CDR1* was assayed using qRT-PCR. Relative expression level data represent the ratio of the mRNA level detected in BVGC89 to that detected in BVGC7. (B) Expression of ERG pathway genes was determined using qRT-PCR. The relative expression levels were calculated as described for panel A.

Construction of a *camPr-ERG1* fusion gene-containing strain (BVGC89) gave rise to a surprising behavior. Even though the addition of camphor to BVGC89 led to a growth defect, expression of the *camPr-ERG1* gene was nearly 8-fold higher than that of wild-type *ERG1* (Fig. 6A) prior to camphor addition. Similarly, both the *PDR1* and *CDR1* levels were also elevated prior to camphor addition. These data indicate that this high-level expression of *ERG1* mRNA (and presumably of Erg1 protein) caused an issue with the ergosterol biosynthetic pathway that was sufficient to activate *PDR1* and *CDR1* expression.

Assays of the mRNA levels of *ERG* pathway genes also demonstrated that these loci were activated by the presence of these high levels of *ERG1* mRNA (and presumably of Erg1 protein) (Fig. 6B). In this case, every *ERG* gene assayed exhibited roughly 2-fold or greater induction.

### An alternative signal repressing Erg11 production still led to Pdr1/*CDR1* activation.

The camphor-repressible promoter experiments supported the view that a reduction in ergosterol biosynthesis led to the activation of *PDR1* and *CDR1* transcription. However, as has been demonstrated for doxycycline and fluconazole (19, 20), it is possible that the presence of camphor interacts with and influences azole resistance and possibly *PDR1* and *CDR1* gene expression.

**FIG 7:**
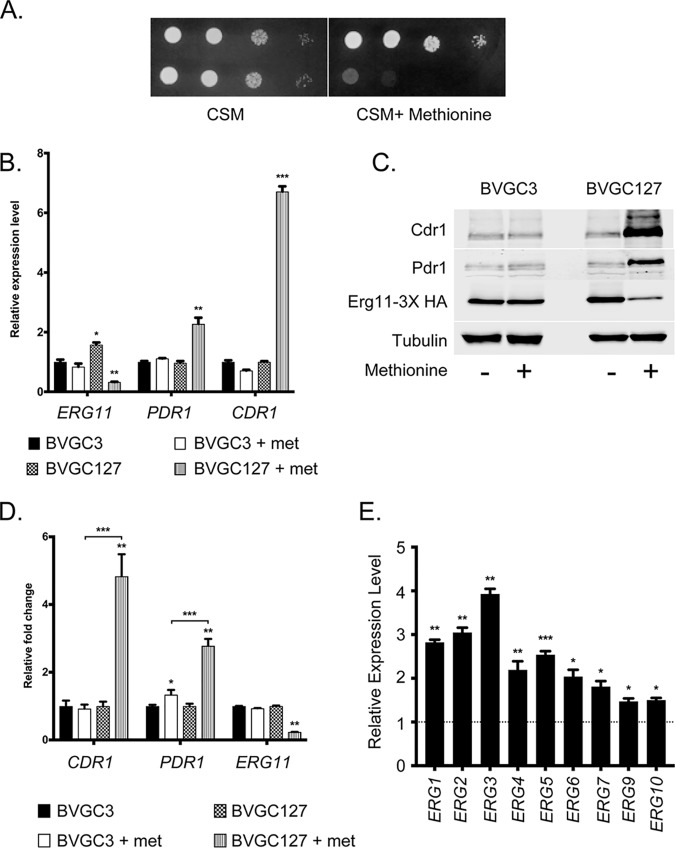
Methionine-dependent repression of *ERG11* transcription induces *PDR1* and *CDR1*. (A) Strains containing the *ERG11*-3× HA allele under the control of the *ERG11* promoter (BVGC3) or regulated by the *MET3* promoter (BVGC127) were grown to the mid-log phase and placed on media lacking methionine (CSM) or containing 1 mM methionine (CSM + Methionine). Plates were incubated at 30μC and then photographed. (B) Overnight cultures of the strains described in the panel A legend were diluted into fresh media containing or lacking methionine and grown for 6 h to regulate *MET3-ERG11* transcription. Cultures were harvested and used for qRT-PCR analysis for the indicated genes. (C) BVGC3 and BVGC127 were grown as described for panel B, but whole-cell protein extracts were prepared and levels of the proteins of interest assessed by Western blot analysis. (D) Quantitation of Western blotting performed as described for panel C. met, methionine. (E) Results of analysis of *ERG* pathway genes by qRT-PCR. The dashed line indicates mRNA levels in BVGC127 cultures grown with no methionine, while the bars denote the mRNA levels in this strain grown for 6 h in the presence of methionine.

To confirm that loss of *ERG11* expression was directly responsible for induction of *PDR1* and *CDR1*, we generated a second repressible system for control of *ERG11* expression. We replaced the *ERG11* promoter with that of the methionine-repressible *MET3* gene. This *MET3Pr*-*ERG11* fusion gene was expected to support growth in a methionine-sensitive manner, as addition of methionine would repress the *MET3*Pr and, subsequently, *ERG11* transcription. The native *ERG11* promoter present in the *ERG11*-3× HA allele in BVGC3 was replaced with *MET3*Pr to create BVGC127. Appropriate transformants were then grown to the mid-log phase, and the ability to support growth on synthetic media with or without methionine was tested (Fig. 7A).

The presence of methionine prevented growth of the *MET3Pr*-*ERG11*-3× HA fusion gene but had no effect on the strain containing a wild-type *ERG11Pr*-*ERG11*-3× HA locus. As this result was consistent with a transcriptional limitation of *ERG11* expression similar to that which we saw earlier with the camphor-off *ERG11* gene, we directly tested whether the induction of *ERG11* repression by the *MET3Pr-ERG11* gene caused by methionine would trigger a response of the Pdr1-*CDR1* circuit. Cells containing the *MET3Pr-ERG11*-3× HA gene were grown overnight and then freshly inoculated into new media with or without with the addition of methionine. After 6 h of growth, the cells were harvested. Both RNA and whole-cell protein extracts were prepared from these cultures.

Levels of mRNA were assayed for *ERG11*, *PDR1*, and *CDR1* by qRT-PCR (Fig. 7B). *ERG11* mRNA levels were strongly repressed during growth in the presence of methionine. In contrast, both *PDR1* and *CDR1* transcription levels rose when the fusion gene was repressed by methionine addition. Steady-state protein levels driven by these genes were also measured by Western blotting using appropriate antibodies (Fig. 7C). Both the Pdr1 and Cdr1 protein levels rose as Erg11-3× HA levels dropped, again consistent with the limitation of Erg11 levels and ergosterol biosynthesis leading to an induction of Pdr1 and *CDR1*. Quantitation of the changes in protein levels revealed that Cdr1 levels rose by approximately 5-fold while Pdr1 levels were elevated 3-fold during methionine repression (Fig. 7D). An identical treatment of an isogenic *ERG11*-3× HA strain under the control of wild-type *ERG11*pr showed no response to methionine addition.

The response of other Erg pathway genes was also examined upon methionine-induced repression of the *MET3-ERG11* fusion gene (Fig. 7E). The *ERG1*-*ERG7* genes were all found to be induced by 2-fold or greater when *ERG11* synthesis was blocked upon methionine addition. These data confirm that, as seen earlier with the camphor-triggered repression of *camP-ERG11*, methionine addition led to ergosterol limitation and subsequent transcriptional induction of the Erg pathway.

### Upc2A provides a transcriptional link between the *ERG* pathway and the Pdr1/*CDR1* regulon.

The data presented above demonstrate an inverse relationship between the levels of transcription of the *ERG11* gene and those of *PDR1* and *CDR1*. The Upc2A transcription factor has been demonstrated to be required for normal regulation of expression of several genes in the Erg pathway, including *ERG11* (23). In addition, isogenic deletion of Upc2A reduced the levels of *PDR1* and *CDR1* induced by azole (15). To confirm this, we constructed an isogenic *upc2AΔ* derivative of the *ERG11*-3× HA strain and subjected it to fluconazole treatment along with BVGC3. This pair of strains was grown to the mid-log phase. These cultures were then split into two aliquots and allowed to grow further in the presence or absence of fluconazole. Whole-cell protein extracts were prepared, and levels of several proteins of interest were analyzed by Western blotting (see Fig. S2 in the supplemental material).

**FIG 8:**
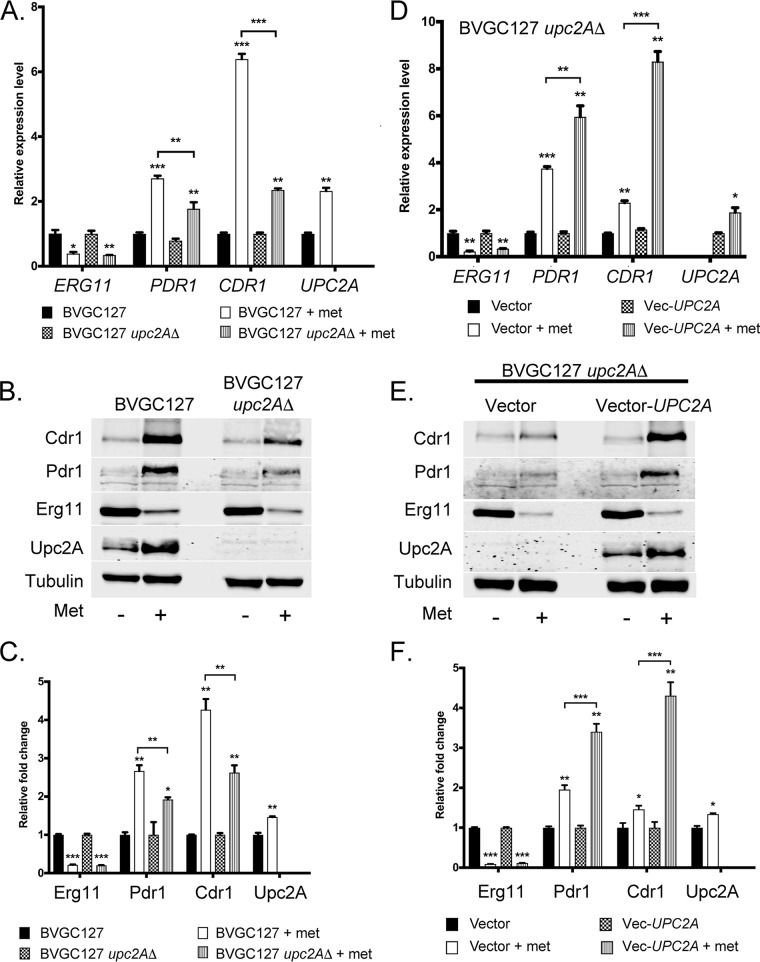
*UPC2A* is required for induction of *ERG11*, *PDR1*, and *CDR1* when *MET3-ERG11* is repressed transcriptionally. (A) Isogenic *MET3-ERG11* strains containing (BVGC127) or lacking (BVGC127 *upc2AΔ*) the *UPC2A* gene were grown in the absence or presence of 20 μg/ml methionine as described above. Total RNA was prepared and levels of the indicated RNAs determined by qRT-PCR. (B) Whole-cell protein extracts were prepared from the strains grown as described for panel A and levels of expression of proteins of interest assessed by Western blot analysis using antisera described previously and a rabbit anti-Upc2A antibody. (C) Quantitation of the Western blotting results presented in panel B. (D) Strain BVGC127 *upc2AΔ* was transformed with a low-copy-number plasmid (Vector) or the same plasmid containing a wild-type copy of the *UPC2A* gene (Vec-*UPC2A*). Transformants were grown as described for panel A and tested for mRNA levels of the indicated genes by qRT-PCR. (E) Whole-cell protein extracts prepared from the transformants prepared as described in the panel D legend were subjected to Western blot analysis using the listed antibodies. (F) Western blotting data determined as described above were quantitated.

The absence of Upc2A led to a decrease in the fluconazole induction of both Pdr1 and Cdr1 while eliminating any detectable increase in the levels of Erg11-3× HA (Fig. S2). This suggested that Upc2A might play a role in control of *PDR1* and *CDR1* transcription that is similar to the role that it plays for *ERG11*. To further explore the connections between Upc2A and the Pdr regulon, we constructed a *upc2AΔ* derivative of the strain containing the *MET3-ERG11*-3× HA fusion gene (BVGC127). As described above, we grew equal aliquots with or without methionine for 6 h. We then analyzed levels of *ERG11*, *PDR1*, *CDR1*, and *UPC2A* mRNA by qRT-PCR (Fig. 8A).

Loss of the *UPC2A* gene decreased the ability of methionine repression of *ERG11* to stimulate expression of both *PDR1* and *CDR1*. We confirmed these data by Western blotting with antibodies against Cdr1, Pdr1, Erg11-3× HA, and Upc2A (Fig. 8B). Note that the addition of methionine lowered levels of Erg11-3× HA but led to elevations of the levels of other four proteins (see Fig. 8C for quantitation).

To confirm that that observed reduction in methionine-induced expression of the Pdr regulon was a consequence of the absence of *UPC2A*, we used a low-copy-number plasmid vector to complement the *upc2AΔ* allele with a wild-type version of this gene. We introduced a *natMX6*-marked vector plasmid containing or not containing a wild-type copy of *UPC2A* into the *MET3-ERG11*-3× HA *upc2AΔ* strain used as described above. Transformants were grown and assayed for methionine-induced expression changes as described above. These data are shown in Fig. 8D to F.

Reintroduction of the wild-type *UPC2A* gene restored normal induction of both *PDR1* and *CDR1* upon methionine-induced repression of *ERG11* transcription. These data support the view that Upc2A must be present to trigger *PDR1* and *CDR1* transcriptional induction upon a reduction in *ERG11* levels.

### Upc2A directly binds to the *PDR1* and *CDR1* promoters.

**FIG 9:**
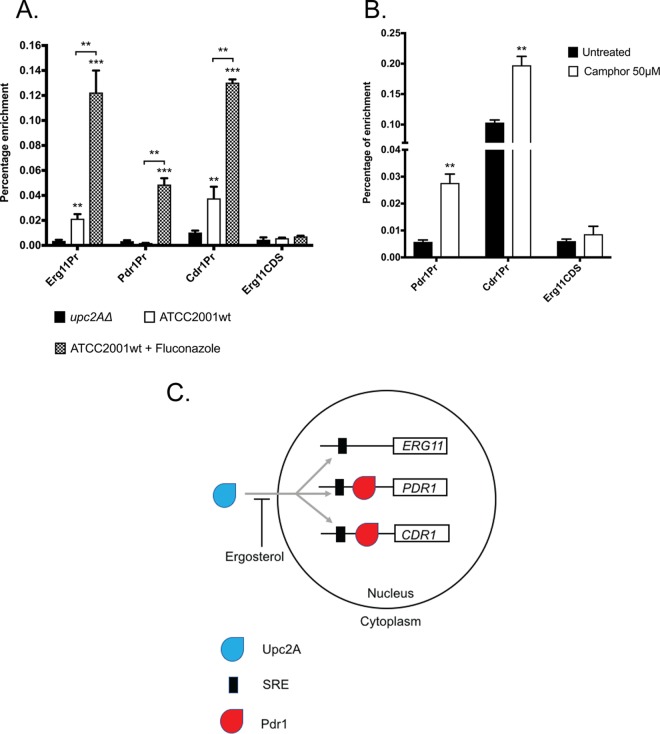
Upc2A binds directly to *ERG11*, *PDR1*, and *CDR1* promoter regions. (A) A wild-type (ATCC2001wt) strain was grown in the absence or presence of fluconazole (ATCC2001wt + Fluconazole), along with an isogenic *upc2AΔ* strain. Total sheared chromatin was prepared from all the strains and immunoprecipitated with anti-Upc2A polyclonal antibody. Immunoprecipitates were analyzed by qPCR using primers specific for the *ERG11*, *PDR1*, and *CDR1* promoters as well as a region of the *ERG11* coding sequence (CDS) as a negative control for enrichment. Percentages of enrichment were calculated by comparing the ratios of each PCR product produced using immunopurified chromatin to that produced in total chromatin. (B) A strain containing the camphor-off allele of *ERG11* was grown in the presence or absence of camphor as indicated. Total chromatin was isolated, sheared, and then immunoprecipitated using the anti-Upc2A antibody. Percentages of enrichment of each PCR product were calculated as described for panel A. (C) Model for the coordinate control of *ERG11*, *PDR1*, and *CDR1* transcription by Upc2A. Based on its striking sequence conservation with S. cerevisiae Upc2, we hypothesize that in the presence of ergosterol, the majority of Upc2A is excluded from the nucleus. Upon limitation of ergosterol, this inhibition is relieved, and Upc2A moves to the nucleus and activates target genes.

Since Upc2A was required for normal transcriptional induction of *PDR1* and *CDR1* upon Erg11 depletion, we wanted to determine if this effect might be a direct one that is associated with Upc2A binding to these promoters *in vivo*. To test this idea, we used the rabbit polyclonal anti-Upc2A antibody that we had developed to perform chromatin immunoprecipitation (ChIP) experiments and probed associations of Upc2A with the *ERG11*, *PDR1*, and *CDR1* promoters (Fig. 9A). ChIP experiments were done in the presence and absence of fluconazole as well as with a *upc2AΔ* strain as a negative control.

Strikingly, Upc2A was found on both the *PDR1* and *CDR1* promoters. A significant association of Upc2A with *CDR1* was seen prior to fluconazole treatment, while Upc2A binding to the *PDR1* promoter was detectable only after azole challenge. Fluconazole-inducible Upc2A binding was also detected on the ERG11 promoter, but Upc2A was found on *ERG11* even with no azole drug present. These data indicate that Upc2A binds to all three of these promoters and is likely to act as a direct transcriptional regulator of these genes.

Having established the presence of inducible Upc2A binding to promoters of interest after azole challenge, we wanted to determine if binding was also seen when we limited production of *ERG11* using our camphor-off *ERG11* fusion gene described earlier. The strain containing the *camP-ERG11* gene was grown in the presence and absence of camphor as described earlier. ChIP using the anti-Upc2A antibody was carried out on these samples, and the levels of *PDR1* and *CDR1* promoter DNA recovered in these reactions were quantitated (Fig. 9B).

Repression of the camphor-regulated *ERG11* gene led to induction of Upc2A binding to both *PDR1* and *CDR1*. Again, there was a significant Upc2A association with the *CDR1* promoter, but not with the *PDR1* promoter, in the absence of *ERG11* repression. These data support the view that limitation of Erg11 activity, mediated either by the presence of azole drugs or by repression of the camphor-off *ERG11* gene, triggers Upc2A binding to target genes (Fig. 9C; discussed below). Upc2A provides a direct connection between ergosterol biosynthesis and expression of genes involved in azole resistance.

## DISCUSSION

C. glabrata is an emerging pathogen of increasing importance in infectious disease, certainly due in part to its proclivity to develop azole resistance. Gain-of-function (GOF) mutations in the *PDR1* gene have been well established to provide the primary means of developing azole resistance through the overproduction of target genes like *CDR1* (21, 22). In C. albicans, an important azole resistance mechanism is provided by substitution mutations with the Erg11 enzyme, the target of azole drugs (reviewed in reference 23), as well as GOF mutations in transcriptional regulators like Tac1 (24) and Mrr1 (25). In both of these *Candida* species, changes in expression of ABC transporter-encoding genes and *ERG* pathway genes have been considered to be due to two separate transcriptional circuits. Additionally, direct experiments in C. glabrata have provided evidence that azole drugs can directly bind to and stimulate activity of Pdr1 in this yeast (13).

The data reported here support two additional modifications to our current understanding of azole resistance in C. glabrata. First, by genetically limiting Erg11 activity, we can trigger induction of Pdr1 and its target gene *CDR1*. This occurs in the absence of any azole drug. Clearly, azole drugs may still act as direct inducers of Pdr1 activity but Pdr1 activation can also occur in response to a reduction in function of the ergosterol biosynthetic pathway. Second, we identify a direct connection between transcriptional control of the *ERG* pathway and expression of both *PDR1* and *CDR1* that is manifested by the function of the Upc2A transcription factor. Together, these data argue for the presence of a physiological link between expression of genes producing plasma membrane-localized ABC transporter proteins and the major plasma membrane sterol component ergosterol that extends beyond the presence of the azole drugs (Fig. 9C).

There is precedent for the idea of direct transcriptional cross talk between *ERG* genes and ABC transporter loci in at least two other organisms. Chromatin immunoprecipitation with microarray technology (ChIP-chip) experiments carried out on C. albicans Upc2 detected binding of this important *ERG* gene regulator to the *CDR1* promoter in this yeast (26). Analysis of the Aspergillus fumigatus transcription factor AtrR indicated that this Zn_2_Cys_6_-containing regulator could directly bind to the promoters of both *cyp51A* (*ERG11* homologue) and *abcG1* (*CDR1* homologue) (27, 28).

The work reported here provides a further understanding of the potential connection between levels of ergosterol pathway function and expression of the Pdr1 regulon in C. glabrata. As mentioned above, this yeast frequently acquires azole resistance and represents an emerging clinical complication. We used two very different signals to induce a limitation of *ERG11* production to argue that this is sufficient for induction of Pdr1/*CDR1*. Previous experiments have demonstrated an unexpected synergy between doxycycline and fluconazole (19). Doxycycline is routinely used as a means to artificially regulate gene expression (29), but the interaction between it and fluconazole complicates interpretations in regard to homeostasis during challenge by this azole drug. We used both a natural product (camphor) and the amino acid methionine as artificial signals to repress *ERG11* transcription with the intent of avoiding any complicating interactions with fluconazole or the genes that mediate resistance to this drug. The two compounds had the same effects of repressing *ERG11* synthesis and triggering the elevation of Pdr1 activity and attendant target genes.

Previous experiments in Neurospora crassa also showed that transcriptional repression of *erg11* in this organism also led to induction of *erg* genes as well as the ABC transporter-encoding locus *cdr4* (30), although no transcription factor was linked to this induction. Repression of *erg11* in this system was accomplished by the use of a copper-repressible promoter as well as use of various *erg* pathway mutants. Copper is both an essential nutrient but also a potent inducer of membrane and oxidative stress (reviewed in reference 31) which could lead to unexpected interactions with regulatory systems in place to control membrane biogenesis and maintenance. However, the range of different signals used to repress *ERG11* production (copper, camphor, methionine) makes it less likely that all of these different agents have similar effects that lead to activation of ABC transporter-encoding loci in two very different organisms (filamentous fungus and yeast). The simplest explanation is that limitation of *ERG11* production and ergosterol biosynthesis leads to the activation of both genes involved in production of this important plasma membrane lipid and to proteins that are embedded and function in this lipidic environment, namely, ABC transporters.

Detection of Upc2A binding to both the *PDR1* and *CDR1* promoters provides an attractive model to explain how this physiological circuitry is controlled. Strong evidence that has been provided by work on Upc2 in Saccharomyces cerevisiae indicates that this transcription factor is excluded from the nucleus when ergosterol levels are high (Fig. 9C) (32). Depletion of ergosterol allows Upc2 to move to nuclear target genes and to induce expression of the *ERG* pathway. Based on experiments using green fluorescent protein (GFP)-labeled Upc2 in S. cerevisiae, roughly 30% of the protein is in the nucleus prior to induction using fluconazole (32).

These data fit well with what is seen by ChIP in C. glabrata with respect to Upc2A DNA binding to the three target promoters examined. Both *ERG11* and *CDR1* exhibited significant levels of Upc2A association prior to fluconazole treatment (Fig. 9A), while *PDR1* showed very little Upc2A prior to drug challenge. Importantly, both *PDR1* and *CDR1* were still inducible by fluconazole, even in the *upc2AΔ* strain (Fig. 8A to C). The level of induction was lower than that seen in a strain with intact *UPC2A*, consistent with this factor playing a role in fluconazole activation of expression of these two genes. It is important that loss of Upc2A has a number of cellular consequences, including a reduction in ergosterol levels, lower baseline expression of *ERG* genes, and a defective response to anaerobic conditions (15, 33). This spectrum of phenotypes indicates that loss of Upc2A has physiological sequelae that could impact other regulatory circuits such as those represented by Pdr1/CDR1. We are presently mapping the sterol response element (SRE) for Upc2A in both the *PDR1* and *CDR1* promoters in order to more specifically examine the consequences of loss of Upc2A binding for activation of these two promoters.

Evidence for potential coregulation of Upc2 function and Pdr1 function was also provided by the analysis of SAGA components in S. cerevisiae cells that lacked the *ERG3* gene (34). Proteomic analyses of proteins that copurified with Spt7-TAP (SAGA component) led to the detection of both Pdr1 and Upc2 but only in samples derived from *erg3* mutant strains. These findings are consistent with our detection of Upc2 recruitment to the *PDR1* promoter such as happens only in the presence of an inducing signal like fluconazole or *ERG11* transcriptional repression (Fig. 9). These findings in S. cerevisiae and C. glabrata suggest that analyses of factors binding to target promoters that are done in the absence of induction face the risk of missing important interactions. Additionally, while we do not yet know the precise location of the SRE in the *PDR1* promoter, two Pdr1 response elements (PDREs) have been mapped to this region (16, 35). This presents the possibility that Pdr1 binding to these PDREs may impact Upc2A recruitment to this region. This regulatory interaction may play a previously undetected role in the control of expression of both *PDR1* and, ultimately, its suite of target genes.

## MATERIALS AND METHODS

### Media, plasmids, and strains.

C. glabrata was grown in rich YPD medium (1% yeast extract, 2% peptone, 2% glucose) or under amino acid-selective conditions in complete supplemental medium (CSM) (Difco yeast nitrogen extract without amino acids, amino acid powder from Sunrise Science Products, 2% glucose). All solid media contained 1.5% agar. Nourseothricin (Jena Bioscience, Jena, Germany) was supplemented to liquid CSM media at 50 μg/ml and to a CSM agar plate at 200 μg/ml to select for C. glabrata strains containing the pBV133 vector (36) and its derivative. The *MET3* promoter is repressed by the presence of methionine (Fisher Biotec, Wembley, Australia) in the media as previously shown (37). Liquid CSM lacking methionine was used to induce expression, whereas addition of methionine at 20 μg/ml was used to repress promoter activity. Methionine (1 mM) was used to supplement CSM agar plates. All strains used in this study are listed in Table 1.

**TABLE 1:** Strains used in this study^*a*^

Strain	Parent	Genotype or description
BVGC3	ATCC 2001	*ERG11-*3× HA
BVGC7	BVGC3	HO::*ADH1Pr-camR*-VP16
BVGC9	BVGC7	HO::*ADH1Pr-camR*-VP16 *camO*- *CYC1Pr-ERG11-*3× HA
BVGC85	BVGC3	*ERG11-*3× HA, *upc2A*Δ
BVGC89	BVGC7	HO::*ADH1Pr-camR*-VP16 *camO*- *CYC1Pr-ERG1-*3× HA
BVGC113	BVGC7	HO::*ADH1Pr-camR*-VP16 *camO*- *CYC1Pr-ERG2-*3× HA
BVGC125	BVGC7	HO::*ADH1Pr-camR*-VP16 *camO*-*CYC1Pr-ERG3-*3× HA
BVGC127	BVGC3	*MET3Pr-ERG11-*3× HA
BVGC130	BVGC127	*MET3Pr-ERG11-*3× HA, *upc2A*Δ

a All listed strains are from this study.

### C. glabrata transformation.

Cell transformations were performed using a lithium acetate method (38). After being heat shocked, cells were either directly plated onto selective agar plates (for auxotrophic complementation) or grown at 30μC at 200 rpm overnight (for dominant-drug selection). Overnight cultures were then plated on YPD or CSM agar plates supplemented with 50 μg/ml or 200 μg/ml of nourseothricin, respectively. Plates were incubated at 30μC for 24 to 48 h before individual colonies were isolated and screened by PCR for correct insertion of the targeted construct.

### Plasmid construction.

All constructs used for homologous recombination into the chromosome were constructed in a pUC19 plasmid vector (New England Biolabs, Ipswich, MA). PCR was used to amplify DNA fragments and Gibson assembly cloning (New England Biolabs, Ipswich, MA) to assemble fragments together. Sequences of the repeated influenza hemagglutinin epitope tag (3× HA) along with S. cerevisiae *ADH1* terminator (Sc*ADH1*tr) and *HIS3MX6* were PCR amplified from the pFA6a-3HA-His3MX6 plasmid (39). This tag element was inserted in place of the stop codon of *ERG11*. The camphor transactivator (camR-VP16), which was under the control of the Sc*TDH1* promoter (Sc*TDH1*pr) and Sc*STR1* terminator (Sc*STR1*tr), was amplified from the pSIB619 plasmid (40). It was then combined with the S. cerevisiae codon-optimized form of the nourseothricin resistance gene and fragments from the immediate upstream/downstream regions of the stop codon of the HO gene. The camphor promoter (*camP*), which included Sc*ADH1* terminator followed by the multiple copies of the camphor repressor operator element (camO) attached to the *CYC1* minimal promoter, was amplified from pSIB426 (40). It then was combined with the wild-type version of S. cerevisiae *LEU2* (Sc*LEU2*) and with fragments from the upstream/downstream regions of the target gene start codon. The C. glabrata *MET3* promoter (*MET3*pr) was amplified from the pBV133 plasmid (36) and assembled with the upstream/downstream regions of the start codon of the *ERG11* gene and wild-type Sc*LEU2*. The *UPC2A* deletion construct was made by assembling the recyclable cassette from pBV65 (14) and fragments from the immediate upstream/downstream regions of *UPC2A*. A plasmid-borne copy of wild-type *UPC2A* was generated by digesting the pBV133 plasmid (36) with SacI and EcoRI (New England Biolabs, Ipswich, MA). The wild-type *UPC2A* gene was inserted via Gibson assembly with 1 kb of 5′ flanking DNA and 0.25 kb of 3′ flanking DNA along with the open reading frame.

### Camphor and methionine sensitivity assay.

Cells were grown in YPD medium to mid-log-phase. Cultures were then serially diluted and spotted onto YPD agar plates containing 50 μM camphor (Sigma-Aldrich, St. Louis, MO). In some experiments, the YPD medium was supplemented with 20 μg/ml ergosterol (Sigma-Aldrich, St. Louis, MO) or Tween 80/ethanol (1:1) as the vehicle control. Ergosterol (2 mg/ml) was made in Tween 80/ethanol (1:1). To test strains containing *MET3*pr, overnight cultures were serially diluted and freshly inoculated directly onto a CSM agar plate supplemented with 1 mM methionine. All agar plates were incubated at 30μC for 24 to 48 h before imaging was performed.

### Total sterol estimation.

Cell total sterol was extracted and measured as previously described (41). In short, cell pellets were lysed in 25% alcoholic potassium hydroxide at 90μC for 2 h. Total sterol was detected by spectrophotometric scanning between the wavelengths of 240 nm and 300nm. The presence of ergosterol in the extracted sample resulted in a four-peak curve with peaks located at approximately 262, 270, 281, and 290 nm.

### Quantification of transcript levels by RT-qPCR.

Total RNA was extracted from cells by extraction using TRIzol (Invitrogen, Grand Island, NY) and chloroform (Fisher Scientific, Hampton, NH) followed by purification with RNeasy minicolumns (Qiagen, Redwood City, CA). RNA was reverse transcribed using an iScript cDNA synthesis kit (Bio-Rad, Des Plaines, IL). Assay of RNA via quantitative PCR (qPCR) was performed with iTaq universal SYBR green supermix (Bio-Rad, Des Plaines, IL). Target gene transcript levels were normalized to transcript levels of 18S rRNA.

### Antibodies and Western blotting.

Mouse Anti-HA antibody was purchased from Thermo Scientific (Waltham, MA), and IRDye secondary antibodies were purchased from Li-COR (Lincoln, NE). Mouse anti-tubulin antibody was obtained from the Developmental Studies Hybridoma Bank, created by the NICHD of the NIH and maintained at The University of Iowa, Department of Biology. To produce the anti-Cdr1 polyclonal antibody, the 169 N-terminal amino acids of *CDR1* were PCR amplified and cloned in frame as a NcoI/SacI fragment downstream of the 6×-His tag in pET28a+ (EMD Millipore Inc.). Expression was carried out in Escherichia coli BL21(DE3) cells (Thermo Scientific, Waltham, MA). Transformants were grown to the log phase and induced with 1 mM isopropyl-β-d-thiogalactopyranoside (IPTG) for 90 min at 37μC. Cell lysates were prepared using a French press. Protein purification was accomplished using Talon metal affinity resin (TaKaRa Bio USA, Inc.) as described by the manufacturer. Protein fractions were analyzed by staining them with Coomassie blue and by Western blotting using His-specific antibodies. The purified proteins were then dialyzed against phosphate-buffered saline overnight (PBS) and then lyophilized and sent to Pacific Immunology (Ramona, CA) for injection into rabbits to generate polyclonal antibodies. Antiserum generated from these rabbits was received and tested for immunoreactivity against C. glabrata cell lysates. The antiserum was then affinity purified against the purified protein using AminoLink Plus coupling resin (Thermo Scientific, Inc.) according to the manufacturer’s instructions. All antibodies were validated by Western blotting against an appropriate isogenic deletion strain (see Fig. S1 in the supplemental material). In the case of Upc2A, the 200 N-terminal amino acids were amplified and cloned as a NcoI/SacI fragment downstream of the 6×-His tag in pET28a+ (EMD Millipore, Inc.) and transformed into the bacterial expression strain BL21-CodonPlus (Agilent, Santa Clara, CA). Western blotting of C. glabrata whole-cell protein extracts was performed as previously described (42), and extracts were prepared using NaOH/β-mercaptoethanol-based lysis. All Western blot experiments were performed in triplicate, and the results were quantitated using Odyssey software and are presented as averages of these determinations with standard errors.

10.1128/mBio.00934-19.1 FIG S1 Characterization of polyclonal antibodies against Cdr1 and Upc2A. (A) Isogenic wt and *cdr1Δ* cells were grown to the mid-log phase, and whole-cell proteins were extracted and then analyzed by Western blotting using affinity-purified anti-Cdr1 antisera. Molecular mass standards are indicated in kilodaltons on the left; the position of the 170-kDa Cdr1 is shown by the arrow. (B) Whole-cell protein extracts were prepared from isogenic wt and *upc2AΔ* cells. Extracts were analyzed by Western blotting as described above, but affinity-purified anti-Upc2A antisera were used to develop this blot. Molecular mass standards are shown on the left, and the position of Upc2A is indicated by the arrow. Download FIG S1, TIF file, 0.6 MB. Copyright © 2019 Vu et al. 2019 Vu et al. This content is distributed under the terms of the Creative Commons Attribution 4.0 International license.

10.1128/mBio.00934-19.2 FIG S2 Fluconazole induction of Pdr1, Cdr1, and Erg11-3× HA requires the presence of the Upc2A transcription factor. (A) Isogenic BVGC3 and BVGC3 *upc2AΔ* cells were grown to the mid-log phase and then treated with 20 μg/ml fluconazole (+) or allowed to continue to grow (−) for 3 h. Cultures were harvested, whole-cell protein extracts were prepared, and levels of the indicated proteins were assayed by Western blotting. (B) Quantitation of the Western blotting results presented in panel A. The presence of fluconazole is indicated as “+ FLC.” Download FIG S2, TIF file, 0.6 MB. Copyright © 2019 Vu et al. 2019 Vu et al. This content is distributed under the terms of the Creative Commons Attribution 4.0 International license.

### Chromatin immunoprecipitation and qRT-PCR.

Protein-DNA cross-linking was performed with 1% formaldehyde (Sigma-Aldrich, St. Louis, MO) for 15 min at room temperature with gentle shaking. The reaction was stopped with 250 mM glycine and with 15 min of shaking at room temperature. About 5 × 10^8^ cells were then pelleted, washed with PBS, and subjected to glass bead lysis or stored at −80μC. Cell pellets were resuspended in 600 μl of lysis buffer (50 mM HEPES [pH 7.5], 150 mM NaCl, 1 mM EDTA, 1% Triton X-100, 0.1% deoxycholate, 0.1% SDS, 1 mM phenylmethylsulfonyl fluoride [PMSF], 1× fungal proteinase inhibitor cocktail) and lysed with 1 ml of 0.5-mm-diameter glass beads for 10 min (5 intervals of 2 min with 30 s cooling between intervals) at 4μC. Samples were then vortexed and split into 3 AFA Fiber Pre-Slit Snap-Cap (6 × 15 mm) MicroTUBEs (Covaris) (130 μl of sample per tube). Chromatin was sheared with an E220 focused ultrasonicator (Covaris) under the following conditions: peak incident power (W), 175; duty factor, 10%; number of cycles per burst, 200; treatment time, 780 s; temperature, 7μC; sample volume, 130 μl (in the presence of E220 intensifier [pn500141]). The contents of the tubes from each sample were then pooled and centrifuged at 10,000 × *g* for 5 min at 4μC. The supernatant was transferred into a new tube, and 20 μl was reserved as an input control fraction to verify sonication and as a control for chromatin immunoprecipitation (ChIP) and qPCR. The sheared chromatin was incubated with rabbit polyclonal anti-Upc2A antibody (1:50 dilution) for 2 h before being incubated together with 30 μl of washed protein G Dynabeads (Life Technologies) overnight on a nutator at 4μC. Washing, reversal of cross-links, and purification of DNA processed by the use of ChIP were performed as described in reference 43.

Real-time PCR was performed in triplicate for each separate ChIP experiment using primers designed for regions described below, under the following conditions: 1 cycle of 95μC for 30 s followed by 40 cycles of 95μC for 15 s and 56μC for 30 s on an MyiQ2 Bio-Rad machine. A 0.5-μl volume of the DNA processed by the use of ChIP or of input (diluted 20-fold) DNA was used in a reaction mixture with a 20-μl total volume using SYBR green master mix (Bio-Rad) and a 0.4 μM concentration of each primer. The percent input method was used to calculate the signal of enrichment of the promoter region for each gene. *ERG11*, *CDR1*, and *PDR1* promoters were analyzed with primers specifically targeting the *ERG11* promoter (−561 to −694 relative to the ATG as +1), *CDR1* promoter (−476 to −665), and *PDR1* promoter (−551 to −651) regions. A region of *ERG11*, located within the coding sequence (939 to 1042), was used as a negative control.

### Statistics.

The Student *t* test was used to assess the statistical significance of results of comparisons of samples. Paired conditions were used for comparisons of results from the same isolate obtained under different treatment conditions, while unpaired conditions were used for comparisons of results from isolates obtained under the same treatment conditions (*, *P* < 0.5; **, *P* < 0.01; ***, *P* < 0.001).
