# Supplementary figures and images for: Evidence that Ergosterol Biosynthesis Modulates Activity of the Pdr1 Transcription Factor in Candida glabrata
Source: mBio. 2019 Jun 11;10(3):e00934-19. doi: 10.1128/mBio.00934-19 (PMC6561024; doi:10.1128/mBio.00934-19)

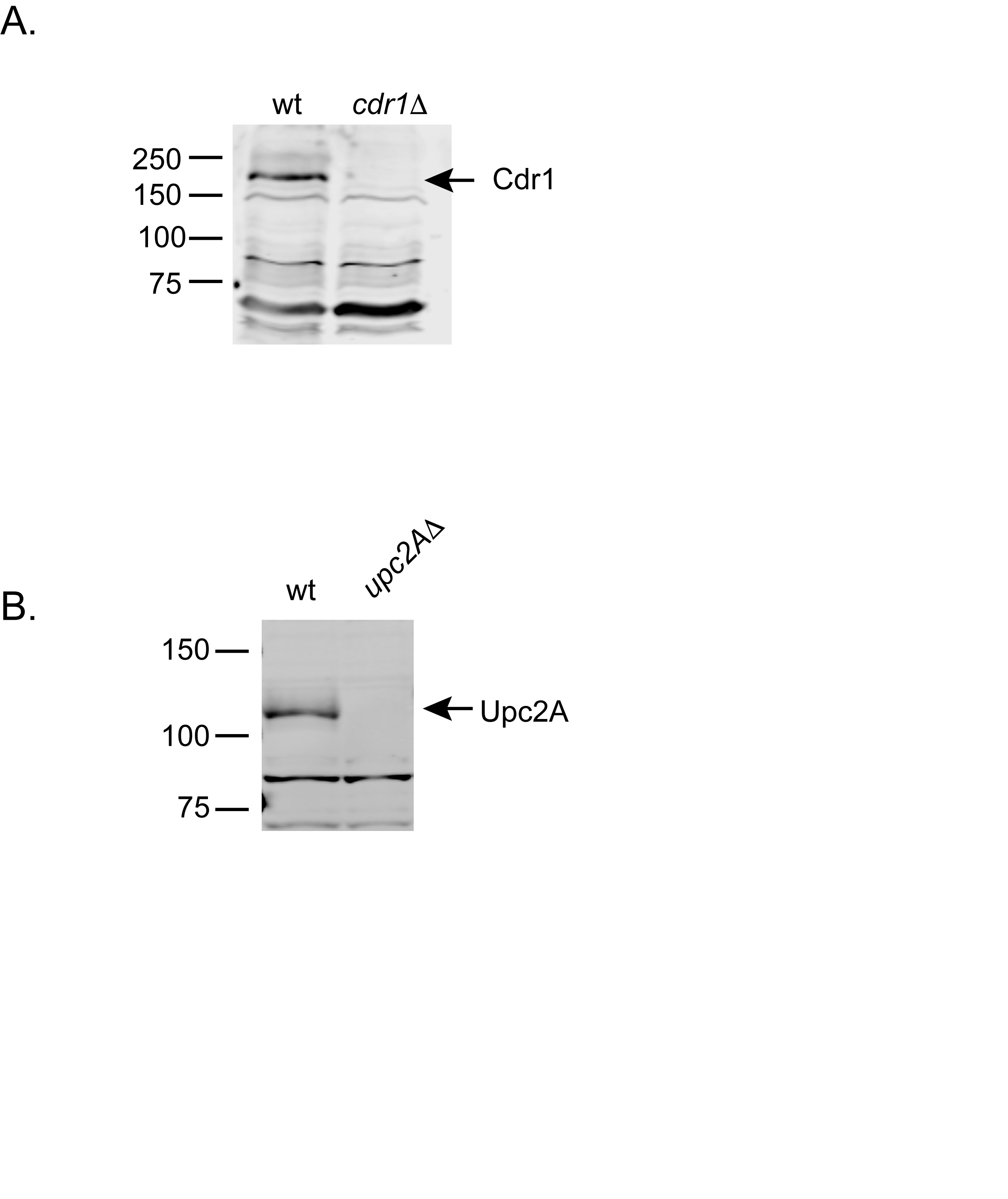

Supplement: FIG S1 [file mBio.00934-19-sf001.tif]

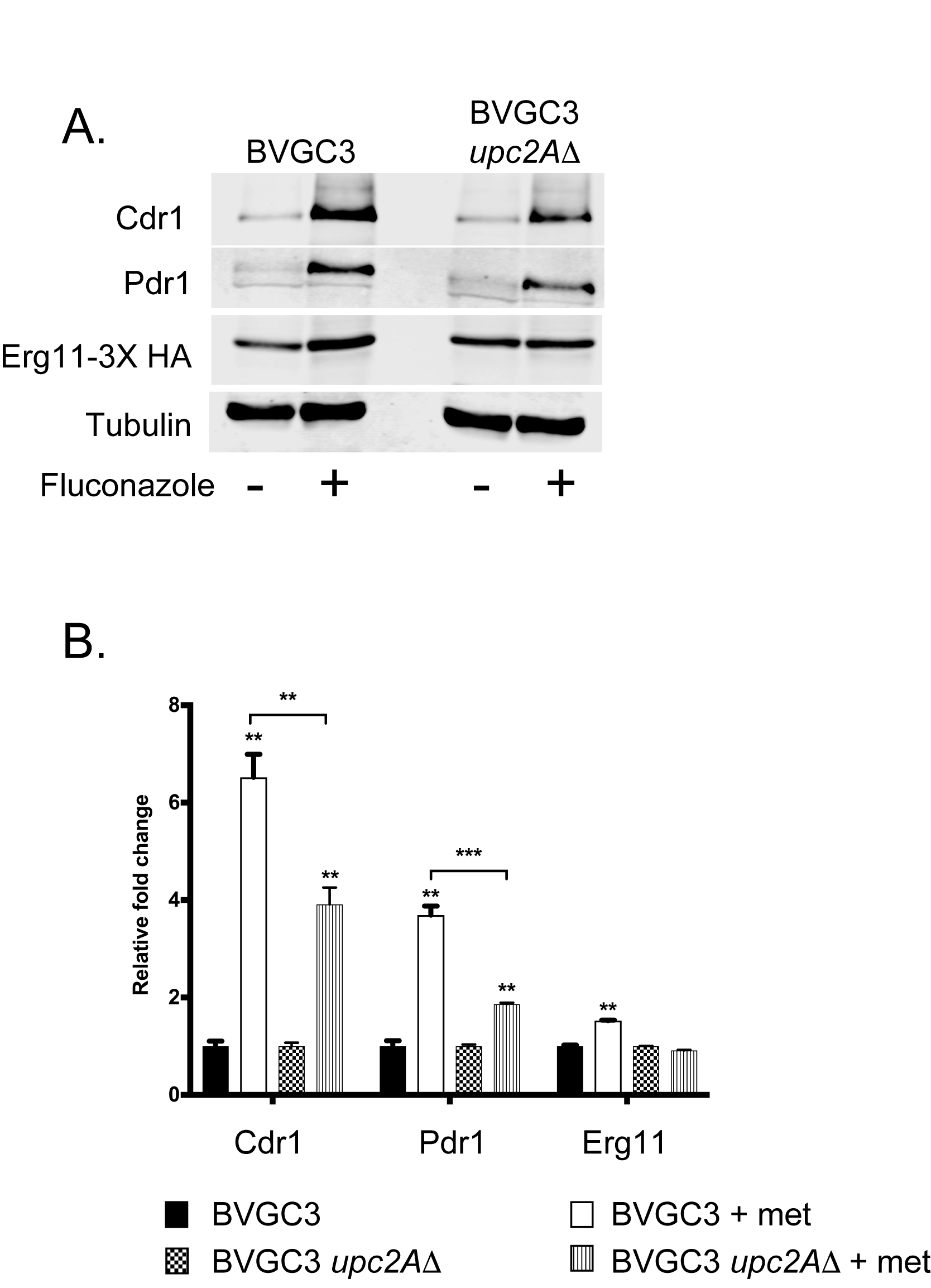

Supplement: FIG S2 [file mBio.00934-19-sf002.tif]
